# Spermidine Feeding Decreases Age-Related Locomotor Activity Loss and Induces Changes in Lipid Composition

**DOI:** 10.1371/journal.pone.0102435

**Published:** 2014-07-10

**Authors:** Nadège Minois, Patrick Rockenfeller, Terry K. Smith, Didac Carmona-Gutierrez

**Affiliations:** 1 Biomedical Sciences Research Complex, University of St. Andrews, St. Andrews, United Kingdom; 2 Institute for Molecular Biosciences, University of Graz, Graz, Austria; Lancaster University, United Kingdom

## Abstract

Spermidine is a natural polyamine involved in many important cellular functions, whose supplementation in food or water increases life span and stress resistance in several model organisms. In this work, we expand spermidine’s range of age-related beneficial effects by demonstrating that it is also able to improve locomotor performance in aged flies. Spermidine’s mechanism of action on aging has been primarily related to general protein hypoacetylation that subsequently induces autophagy. Here, we suggest that the molecular targets of spermidine also include lipid metabolism: Spermidine-fed flies contain more triglycerides and show altered fatty acid and phospholipid profiles. We further determine that most of these metabolic changes are regulated through autophagy. Collectively, our data suggests an additional and novel lipid-mediated mechanism of action for spermidine-induced autophagy.

## Introduction

The understanding of the mechanisms underlying the aging process is of general interest; not least because it opens doors to modulate it and eventually postpone or prevent age-related pathologies and thus improve our health span. Aging research in the last three decades has elucidated a series of mutations in single genes that increase life span in many organisms [1]. In addition, dietary restriction (DR) has advanced to be the most reliable means to increase life span and reduce age-related diseases in many organisms [2], although the latest results from studies in non-human primates are not as promising as expected, at least in regard to life span [3]. Despite these findings, the most convenient method to counteract the detrimental effects of aging would be the simple ingestion of compounds with the ability to do so. In fact, several pharmacological interventions have been shown to hamper age-related diseases and to be beneficial for health and life span. For instance, resveratrol, a naturally occurring phenol, increases the life span of mice kept on a high-fat diet [4]. However, resveratrol does not exert any beneficial effect in healthy organisms [5]. The immunosuppressant drug rapamycin also increases the life span of rodents [5], [6], but shows inconclusive effects in the fruit fly *Drosophila melanogaster* and – being an immunosuppressant - its actual use for humans remains doubtful [7], [8].

Spermidine is a natural polyamine involved in many important molecular processes such as DNA stability, transcription, translation, apoptosis, cell proliferation, differentiation, and survival [9]. Intriguingly, its intracellular level decreases with age [10], [11]. Indeed, a recent study shows that spermidine levels decrease in 60–80 year-old people compared to 31–56 year-olds [12]. However, in that same study levels in 90–106 year-old people were similar to those found in the youngest age group [12], suggesting that maintenance of high spermidine concentration during aging may contribute to longevity. In accordance with this concept, our previous work [13] established that food supplementation with spermidine increases the life span of the yeast *Saccharomyces cerevisiae*, the nematode worm *Caenorhabditis elegans*, the fruit fly *Drosophila melanogaster*, and that of cultured human immune cells. A high-polyamine diet has also been reported to increase the life span and reduce the age-related pathology of a short-lived mouse strain [14]. Spermidine also increases stress resistance in yeast (heat and hydrogen peroxide) [13] and flies (paraquat and hydrogen peroxide) [15], while it decreases markers of age-related oxidative damage in mice [13]. Conversely, the intracellular depletion of spermidine by genetic mutations in the polyamine pathway decreases the life span of yeast [13] and mice [16].

In yeast, worms, flies and Hela cells, spermidine directly induces autophagy [13], [17], the major cellular recycling mechanism, which is induced upon many interventions that increase life span [18], [19]. In fact, we could previously show that spermidine’s beneficial effects on aging are mainly due to the induction of autophagy [13]. Consistently, spermidine does not increase life span in autophagy-mutant yeast, worms and flies [13]. However, we found more recently that autophagy induction is not responsible for all the beneficial effects exerted by spermidine: For instance, the anti-necrotic function of the propeptide of yeast cathepsin D is spermidine-dependent but autophagy-independent [20]. Also, spermidine can still increase resistance to hydrogen peroxide in autophagy-deficient flies [15]. Thus, under certain circumstances additional mechanisms other than autophagy must be responsible for spermidine-mediated cytoprotection.

Metabolic regulation has long been connected to life span modulation: many mutations known to increase life span also alter metabolism and nutrient sensing, for instance mutations in the IIS (Insulin-Like Signalling) pathway [21], the TOR (target of rapamycin) pathway [22], or in sensory systems [23]. Mounting evidence also places lipid metabolism as an important player during aging [24]–[26]. Many interventions that enhance life span, for example, also increase the abundance of triglycerides (TAG), which represents the major lipid store [27], [28]. In fact, the metabolic targets associated to spermidine’s beneficial effects might be multiple. Its molecular structure allows a plethora of different interactions that might go even beyond multiple ones already described [9], [29].

In this work, we expand spermidine’s scope of potential beneficial effects during aging by showing that it improves age-dependent locomotor activity in *D. melanogaster*. Importantly, we additionally uncover an impact of spermidine on lipid composition, which involves an increase in weight and TAG levels as well as alterations in fatty acid and lipid profiles, most of which seem to be autophagy-dependent. These results are the first showing spermidine’s potential to modulate lipid metabolism and suggest a possible involvement of this capability in the substance’s mechanisms to exert beneficial effects during aging.

## Results

### Spermidine improves locomotor performance in aged flies

**Figure 1:**
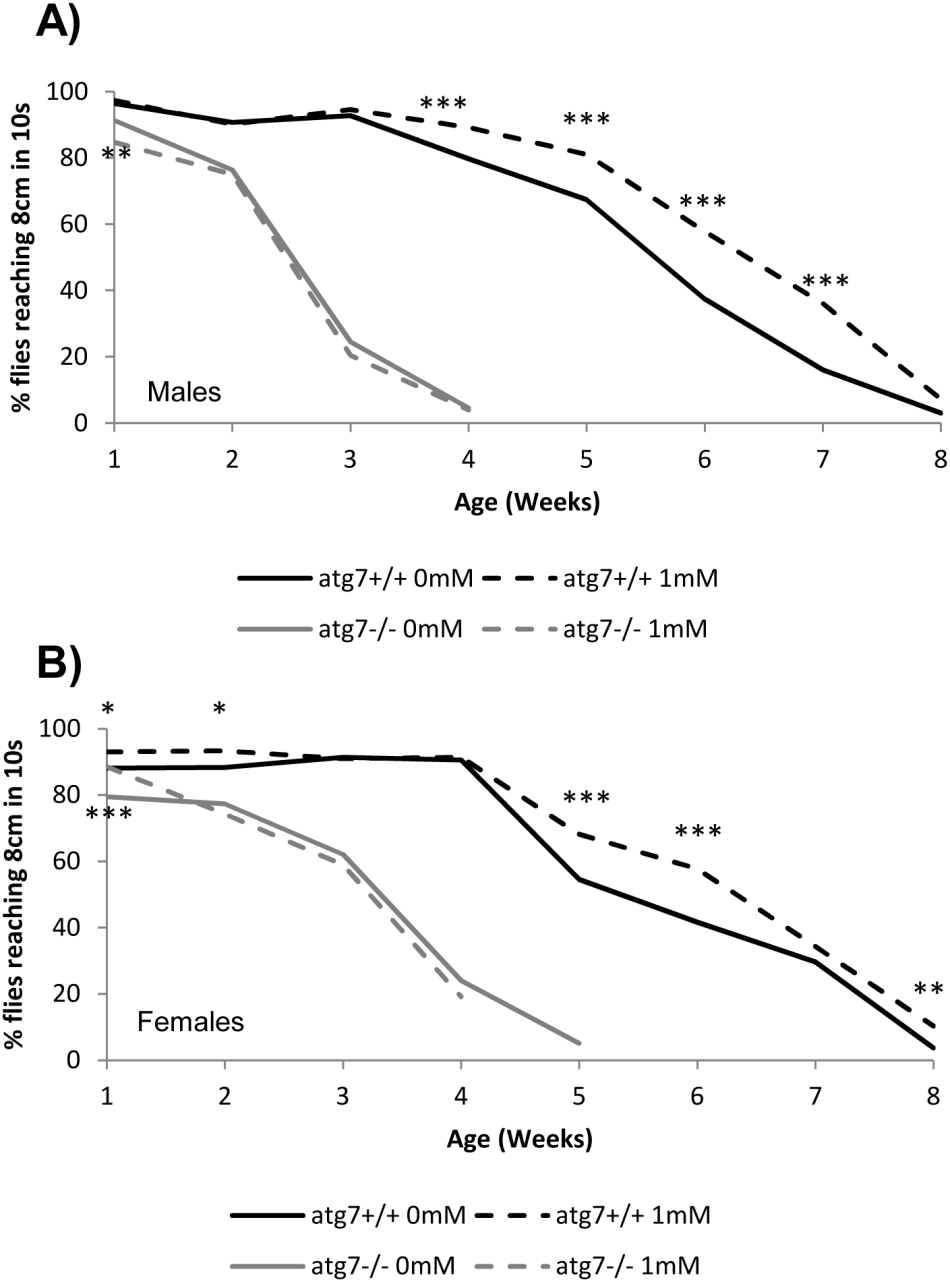
Climbing activity. **A)** Percentage of flies able to climb 8 cm in 10 seconds in wild type and atg7^−/−^ male flies fed 1 mM spermidine or not. 3 independent replicates were monitored (50 flies in each group and replicate). **B)** Percentage of flies able to climb 8 cm in 10 seconds in wild type and atg7^−/−^ female flies fed 1 mM spermidine or not. 3 independent replicates were monitored (50 flies in each group and replicate). *p<0.05; ***p<0.001.

We have previously demonstrated that spermidine-fed flies show an improved survival during aging [13]. We therefore decided to first assess if such improvement might correlate with a decreased incidence of an age-related phenomenon, the progressive decline of locomotor activity. For this purpose, we followed the ability of flies to climb the vertical wall of the vial, in which they were kept. The measurement was done weekly until none of the flies could reach the threshold (8 cm in 10 s). Expectedly, the climbing activity of wild type cells diminished with ongoing age (Fig. 1A) [30], [31]. Spermidine feeding (1 mM) could, indeed, partly rescue this age-dependent decline in both female and male wild type animals (Fig. 1A, B). Importantly, spermidine did not have such effect in loss-of-function mutants for atg7 (*atg7^−/−^)*, a gene essential for autophagy. These animals showed a much faster decline in activity compared with wild type flies and no improvement with spermidine (Fig. 1A, B). Thus, spermidine alleviates age-dependent locomotor impairment in flies in an autophagy-dependent manner.

### Spermidine increases stored lipid content and alters lipid profiles

**Figure 2:**
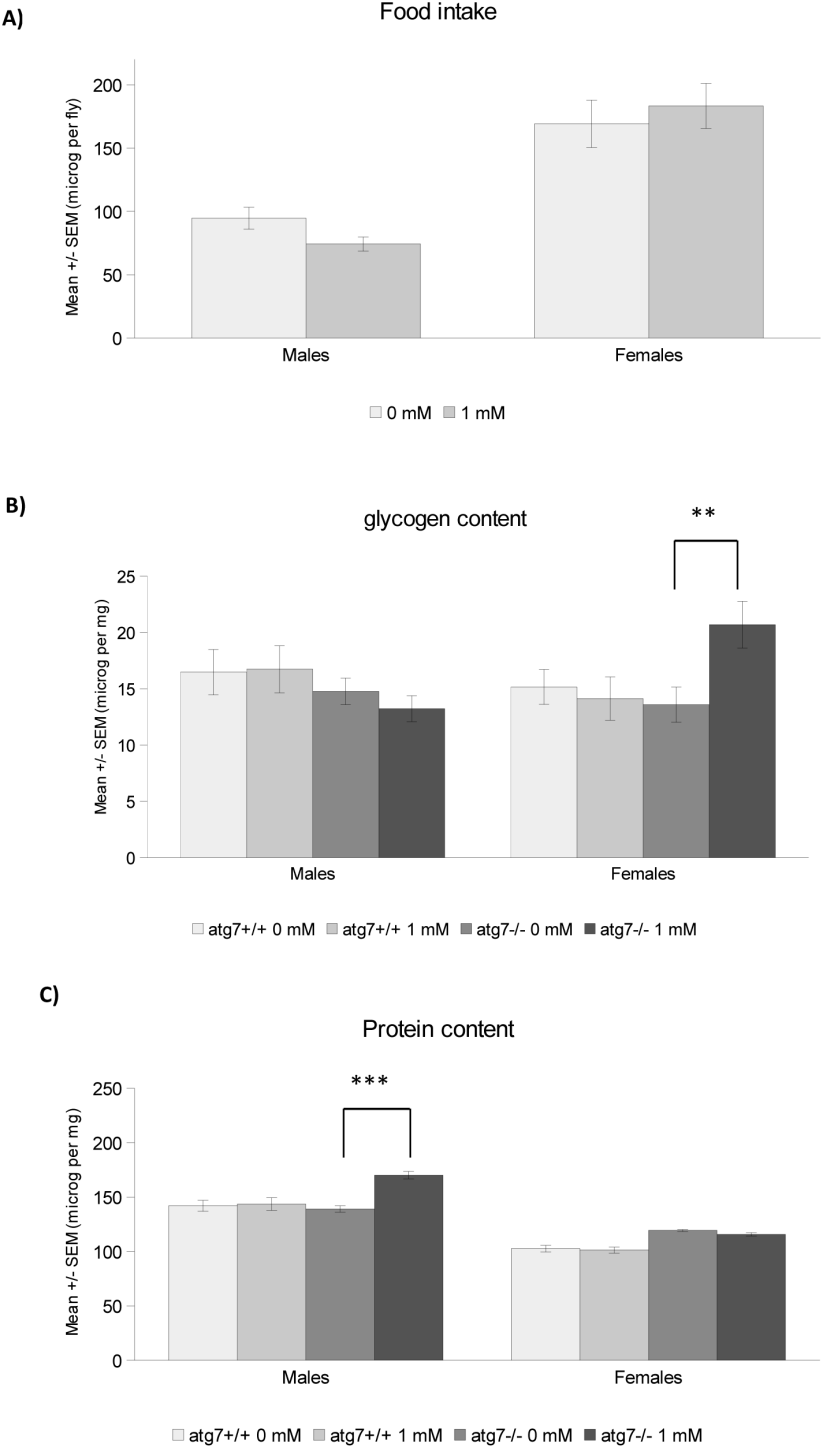
Food intake, glycogen and protein contents. **A)** Mean +/− SEM of food intake in wild type flies of both sexes fed 1 mM spermidine or not as measured by colorimetry (flies fed blue dye and dye intensity measured). 2 independent replicates were monitored (5 or 6 independent samples for each group in each replicate). **B, C)** Mean +/− SEM of glycogen (B) and protein (C) content in wild type or atg7^−/−^ flies of both sexes fed 1 mM spermidine or not as measured by colorimetry (anthrone reaction for B and Bradford reaction for C). 3 independent replicates were monitored (4 or 5 independent samples for each group in each replicate). **p<0.01; ***p<0.001.

**Figure 3:**
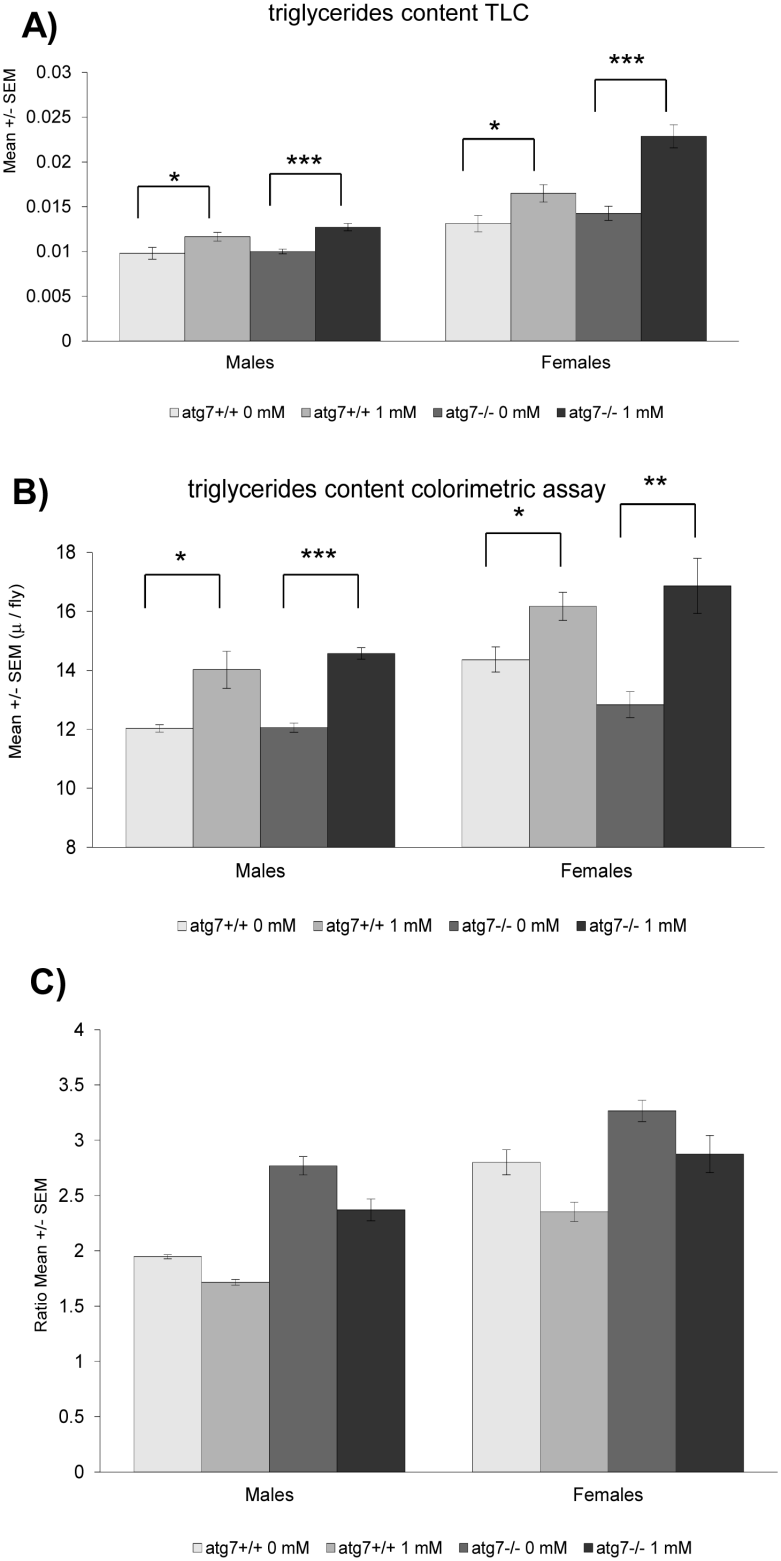
Triglyceride (TAG) content and ratio of saturated over unsaturated fatty acids from TAG. **A, B)** Mean +/− SEM of triglyceride (TAG) content in wild type or atg7^−/−^ flies of both sexes fed 1 mM spermidine or not, measured by thin layer chromatography (A) with 3 independent replicates (4 independent samples for each group in each replicate) or a colorimetric assay kit (B) with 3 independent replicates monitored. *p<0.05; **p<0.01; ***p<0.001. **C)** Ratio (+/−SEM) of saturated over unsaturated fatty acids from TAG in wild type and atg7^−/−^ flies fed 1 mM spermidine or not. 3 independent replicates were monitored for each group.

**Figure 4:**
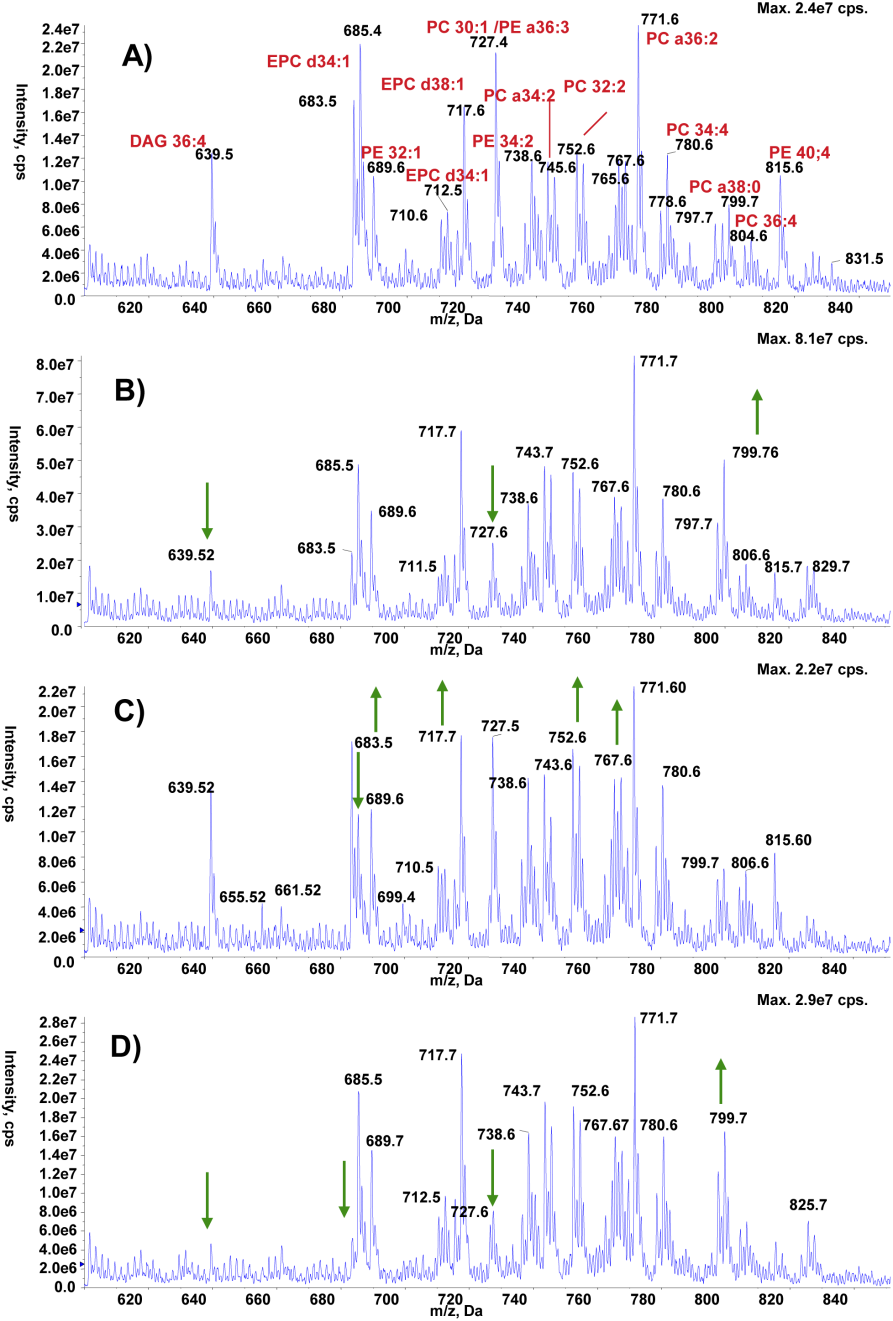
Phospholipid profile of males positive survey. **A–D)** Positive ion ES-MS survey scans (600–850 m/z) of total lipid extracts from male wild type (A, atg7^+/+^) and atg7^−/−^ (B) flies as well as from spermidine-fed male wild type (C, atg7^+/+^) and atg7^−/−^ (D) flies. Arrows indicate significant changes compared to normal flies untreated.

**Figure 5:**
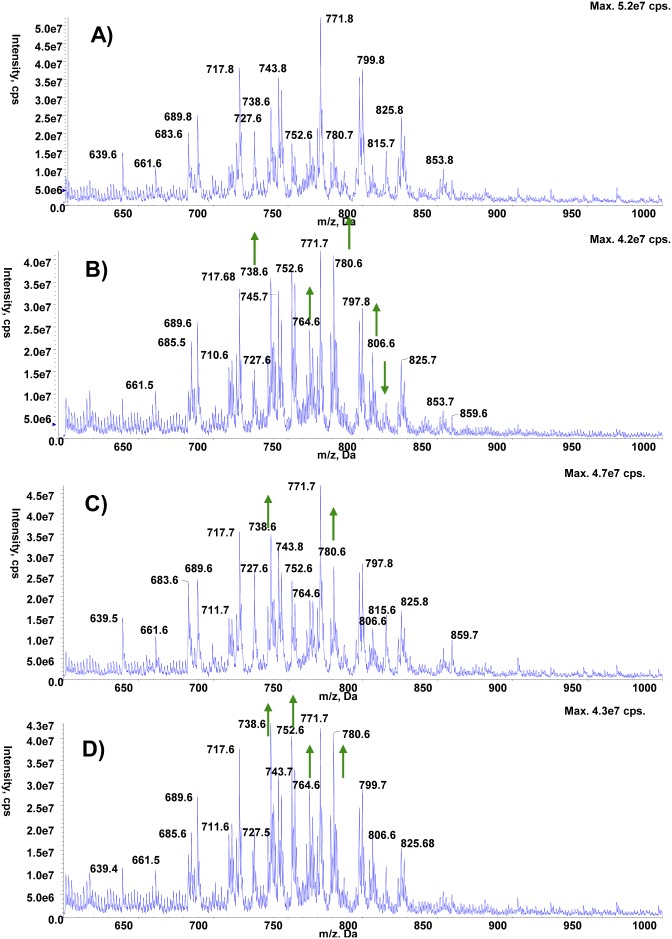
Phospholipid profile of females positive survey. **A–D)** Positive ion ES-MS survey scans (600–1000 m/z) of total lipid extracts from female wild type (A, atg7^+/+^) and atg7^−/−^ (B) flies as well as from spermidine-fed female wild type (C, atg7^+/+^) and atg7^−/−^ (D) flies. Arrows indicate significant changes compared to normal flies untreated.

Age-related decline in motor performance is one of the phenotypes arising from neuronal aging, for which recent evidence suggests an important contribution of changes in the lipid membrane composition [32]. In fact, lipid metabolism seems to be a prominent player during aging [24]–[26] and many life span-enhancing interventions also increase the levels of TAG [27], [28]. We thus decided to analyze the impact of spermidine feeding on fly lipid profiles. Even though treatment of wild type flies with 1 mM spermidine for one week did not affect food intake, glycogen or protein levels (Fig. 2A–C), it increased the animals’ TAG-content as measured both by thin layer chromatography (Fig. 3A) and a colorimetric assay (Fig. 3B). Interestingly, total and TAG-derived fatty acids displayed specific changes in male and female flies, respectively (Fig. S1, S2, Table S1). Of note, the ratio of saturated over unsaturated TAG-derived fatty acids declined upon spermidine treatment (Fig. 3C). Moreover, lipidomic electrospray-mass spectrometry analysis revealed a significant impact of spermidine on the levels of a multitude of lipid species. All lipid species were fully characterized (Table S2). While the phospholipids profile in the negative ion mode survey scans did not show any significant differences between spermidine-treated and untreated animals in both sexes (Fig. S3, S4), the positive survey revealed changes upon spermidine feeding: males displayed increased levels of EPCd38∶1, PC32∶2 and PE38 as well as enhanced saturation of EPCd34 (Fig. 4). In females, spermidine induced an increase in PE34∶2 and PC34∶4 (Fig. 5). Collectively, these findings show that spermidine affects lipid metabolism by virtue of increased TAG levels, altered fatty acids and phospholipids composition.

### Autophagy regulates spermidine-mediated lipidomic changes

Lipid metabolism has recently been described to act at the crossroads of longevity and autophagy [33]. In order to test whether autophagy is connected to spermidine-mediated lipid changes, we analyzed this aspect in autophagy-deficient mutants (*atg7^−/−^).* It should be noted that these mutant flies showed no significant differences on overall lipid levels (Fig. 3A, B), even though they did present some changes in their fatty acid profiles (Table S1). Interestingly, spermidine maintained and even exacerbated its enhancing effect on TAG levels (Fig. 3A, B) as well as on sex-specific changes in total and TAG-derived fatty acids (Fig. S1, S2, Table S1), with female flies showing a higher and male flies a lower overlapping changing TAG-derived fatty acids compared with those observed in wild type flies (Table S1). The decline observed in the ratio of saturated over unsaturated TAG-derived fatty acids was also maintained (Fig. 3C). Thus, the general TAG-increase observed with spermidine is autophagy-independent, although the level of TAG itself may be autophagy-dependent. In contrast, the profile of TAG-derived fatty acids triggered by spermidine seems to depend on functional autophagy. Intriguingly, spermidine failed to induce the effects on phospholipid profiles observed in wild type animals: In *atg7^−/−^* females, no differences whatsoever were observed (Fig. 5) while in *atg7^−/−^* males spermidine decreased the saturation of EPCd34 as opposed to wild type animals (Fig. 4). Altogether, this shows that spermidine exerts its effects on TAG-derived fatty acid and lipid profiles in an autophagy-dependent fashion.

## Discussion

Our previous work has demonstrated the beneficial potential of spermidine in terms of age-related stress resistance and survival during aging [13], [15]. In this study, we expand these findings by reporting a correlation of these benefits with a decreased incidence of the age-related decline in locomotor activity. Interestingly, a putative effect of polyamines on enhanced activity during aging has been previously anecdotally reported for mice fed a high-polyamine diet, which were described to be more active and to keep a thicker coat with age, but these variables were not measured [14]. Furthermore, we had already reported that spermidine rescues the decline in activity of flies under paraquat exposure [15]. In contrast to our results, a recent report showed no protective effects of spermidine on the age-dependent decline of climbing activity in flies [34]. While both studies use a similar protocol to assess climbing activity, we do find a positive effect in both sexes. Two main reasons may account for this discrepancy. Firstly, Gupta et al. used a much smaller sample size: one group of ten flies [34] compared with five groups of ten flies in the present study. Secondly, the percentage of old flies (30 days of age in that study) reaching the threshold is much lower than the percentage we report here at similar ages (between 4 and 5 weeks): around 45% in that report [34] versus approximately 80% in our study. Given the higher sample size and the seemingly more optimal physiological state of the flies used in this study, we rely on the herein reported data in anticipation of further studies that may resolve these varying results.

Age-dependent decline in motor performance has been suggested to be linked to lipid metabolism [32], which in general seems to be connected to the aging process [24]–[26], [35], [36]. Intriguingly, spermidine has been recently identified and characterized as a key factor in the differentiation of preadipocytes into mature adipocytes, and thus in the process of adipogenesis [37], [38]. However, our results cannot answer whether changes in lipid level and composition are the causes for spermidine-mediated locomotor improvement. Locomotion is controlled by two main organs/tissues: brain/neurons and muscles. On the one hand, it is known that spermidine interacts with brain cells: It has anti-inflammatory effects on microglia cells subjected to lipopolyssacharides exposure [39] and spermidine levels are linked to memory [34] as well as to disorders like Alzheimer’s disease [40]. It would thus be no surprise that spermidine improves age-related locomotion by altering neuronal cells or communication between neurons and muscles. On the other hand, the effects of polyamines on muscle function have been much less studied and have focused mainly on smooth muscles, especially heart muscle cells. Anabolic agents have been shown to increase both skeletal muscle mass and polyamines levels, but a causal link between polyamines and muscle growth has not been investigated [41]. Of note, the initiation factor eIF5A, whose synthesis requires spermidine, increases skeletal muscle stem cell differentiation [42]. Since skeletal muscle is one of the tissues most prone to aging (sarcopenia, [43]), it is also equally plausible that spermidine acts directly or indirectly on muscles to improve locomotion in old age.

Our herein presented results suggest that at least some spermidine-mediated beneficial effects might be, indeed, related to lipid changes. For instance, we report that spermidine-fed flies show increased TAG levels, which has often been associated with increased life span [27], [28], [44], [45]. In a similar manner, long-lived Drosophila (upon overexpression of dSir2) show an up-regulation in the expression of genes involved in fat metabolism [46] and, in turn, increased expression of genes related to fatty acid β-oxidation extends life span in flies [47]. In mice, the ability to maintain fat content under dietary restriction (DR) correlates with life span extension upon DR [26]. In light of our findings, we reanalysed our previously published microarray data on the effect of spermidine on gene expression in yeast, focusing on genes related to lipid metabolism (data available at https://www.ebi.ac.uk/arrayexpress/experiments/E-MTAB-912/ [48], previously published in 13). In support of the present results, spermidine alters the expression of many genes involved in lipid metabolism, some being up-regulated (e,g., FAA1 and FAA4, long-chain fatty acyl-CoA synthetases or SKN1, involved in sphingolipid biosynthesis) and some being down-regulated (e.g., PIS1, a phosphatidylinositol synthase or ISC1, inositol phosphosphingolipid phospholipase C), showing that spermidine profoundly regulates the expression of lipid-associated genes.

Whether spermidine-mediated TAG increase can be associated to spermidine-induced life span extension is not clear, since autophagy mutants, in which spermidine fails to trigger longevity [13], display an even higher TAG-content. Thus, the observed TAG increase could be the result of spermidine’s autophagy-independent survival effects [15], [20], or other molecular or organismal consequences of spermidine treatment. Among the diverse lipid species that play a role in metabolism, fatty acids (FFAs), diacylglycerol (DAG), ceramide and cholesterol are considered the ones bearing lipotoxic potential, whereas TAG is generally thought as being a rather inert fat store. Of course, massive TAG deposition in various tissues other than adipose tissue (e.g. muscle, heart, liver) is considered lipotoxic [49], but TAG primarily serves at detoxifying FFAs and DAG than being toxic itself. Consequently, FFAs and DAG steady state levels are very low and they only serve as metabolic intermediates. An increase in TAG as a response to spermidine feeding could thus mean that this detoxification capacity is enhanced. Possibly, autophagy is directly involved in the process of lipid remodeling. A connection of autophagy to lipid turnover has been previously identified [50], but the extent of its interconnection might still be underestimated. Thus, the actual relevance of the herein described TAG-increase upon spermidine supplementation, i.e., an optimal amount for improved fitness, will have to be clarified in future studies.

Besides a higher TAG content, spermidine also altered TAG-derived fatty acids towards a decreased ratio of saturation to unsaturation. Moreover, spermidine triggered profound changes in phospholipid profiles. Among these alterations, the increased saturation of ceramide phosphoethanolamine (CPE - or ethanolamine phosphate-ceramide species) EPCd34 in male flies appears most interesting. CPE is the main sphingolipid in invertebrates [51] and involved in signalling as well as an essential membrane component that provides scaffolding for organisation and stability. In this light, the effects of spermidine on EPCd34 saturation in males may affect signalling and membrane fluidity, which would have important consequences for aging. For instance, mitochondrial membrane fluidity is a critical factor during aging and neurodegeneration [52] and may modulate the response to high oxidative stress deriving from molecular damage. Further experiments will have to address whether there exists a causal relationship between changes in membrane fluidity and the beneficial effects of spermidine. It must be noted that we could not measure any changes in EPCd34 saturation of female flies. However, this particular lipid species is much less abundant in females and here a more fine-tuned modulation might occur that could not be detected in this study. We found that spermidine has qualitatively or quantitatively different effects on lipid levels and composition in males and females. Indeed, male and female flies exhibit different lipid profiles, especially differences in saturation levels in various lipid species [53]. Given the different nature of lipids, it is not surprising that spermidine may affect them differently. This may partly explain the differences of sexes we have observed on other variables upon spermidine treatment, e.g., life span [13], resistance to stress [15] or locomotor activity (this study).

According to our results, the above delineated qualitative and quantitative impact of spermidine on lipid levels and composition partly seems to depend on autophagy. Autophagy deficiency eliminates most of the effects of spermidine on lipid profiles or even reverses them as with the saturation ratio of EPCd34. On the other hand, lack of autophagy exacerbates spermidine-mediated TAG-accumulation, which as discussed above rather suggests that this effect is not associated to spermidine-mediated longevity, which is mainly autophagy-dependent [13]. Nevertheless, some of the other lipidomic changes (saturation ratio and fatty acid and phospholipid profiles) observed in spermidine-treated wild type flies and abrogated in autophagy-mutant animals might be linked to spermidine-induced longevity since the modulation of lipid metabolism has been recently connected to autophagy and longevity [25], [33]. Still, the general role of autophagy on lipid metabolism remains a matter of debate as studies report contrasting effects [50], [54]–[56] and thus needs further investigation. Besides these considerations, it should be noted that spermidine seems to trigger metabolic effects beyond the lipid realm: for instance, while spermidine-treatment did not lead to any changes in protein or glycogen levels in wild type flies, autophagy-mutant females showed a higher glycogen (Fig. 2B) and males a higher protein level (Fig. 2C). This may point towards further metabolic aspects, in which regulatory processes connecting autophagy and spermidine are involved.

It will be interesting to see whether the herein described modulation of lipid level and composition interacts with some of the previously described mechanisms of spermidine-mediated beneficial effects, including general hypoacetylation and phospho-regulatory pathways [13], [57], [58] or PKB/Akt [59], [60] and the MAPK pathway [60]–[63]. In fact, flies knocked-down for Loco, which regulates inhibitory G proteins that in turn regulate MAPK [64], [65], show increased life span and enhanced stress that is associated with an increase in TAG levels compared with controls. These phenotypes are similar to those observed in spermidine-treated flies. Intriguingly, Loco knocked-down flies exhibit decreased cAMP levels, which may represent a molecular link connecting spermidine, MAPK and lipid metabolism and thus be worthy of future studies.

In summary, we here uncover the far-reaching impact of spermidine treatment on lipid levels and composition, which at least in part seems to be autophagy-dependent. It is tempting to theorize that these spermidine-induced changes might be involved in the plethora of beneficial effects that spermidine exhibits during stress and aging. Future studies will have to clarify the causality of the herein described phenomenon in order to establish a further branch of action in spermidine’s extensive impact on cellular metabolism.

## Materials and Methods

### Flies Husbandry

The atg7^−/−^ flies were kindly provided by T. Neufeld (University of Minnesota) [66]. Flies were collected as virgins and were kept unless otherwise stated on a standard cornmeal-sugar-yeast diet at 25°C. Males and females were kept separately. The food was changed twice a week and supplemented or not with spermidine (Sigma, S4139) at a 1 mM final concentration.

### Triglycerides content

The protocol for measurement of triglycerides content by thin layer chromatography is as described in Al-Anzi et al. [67]. In each vial, 20 flies were kept and given spermidine or not for one week, after which the flies were then frozen in liquid nitrogen. Ten flies were homogenized in 250 µl of a 2∶1 chloroform (VWR, 100777C):methanol (Sigma, 34860) solution, and 2 µl were spotted onto a TLC plate (VWR, 1.16835.0001). Each plate was run in 4∶1 hexane (Sigma, 270504):ethyl ether (Fisher Scientific, D/2506/15) until the solvent reached almost the top of the plate. The plate was then dipped in ceric ammonium molybdate (2.5 g ammonium heptamolydbate tetrahydrate (Sigma, 431346), 1 g cerium (IV) sulphate hydrate complex with sulfuric acid (Sigma, 423351), 90 ml water, 10 ml concentrated sulfuric acid (Sigma, 258105). The plates were then dried with a hair dryer and a picture taken with an UVIdoc HD2 system (Uvitec). The area and mean grey value of the triglycerides spots were measured with ImageJ (http://rsbweb.nih.gov/ij/). Lard was run on each plate as a control. Three independent replicates were performed, each with four independent samples for each group. Triglycerides content was also measured using a commercial colorimetric kit (Cambridge Biosciences, Caymen triglyceride colorimetric assay kit 10010303). In each vial, 20 flies were kept and given spermidine or not for one week. Three independent samples were prepared for each group consisting of 10 flies (fresh tissue), which were put in a Eppendorf tube with 200 µl PBS and 750 µl of a 1∶2 chloroform:methanol solution. The flies were homogenized and then transferred into glass vials. The samples were then mixed for 15 minutes on a vortex. A further 250 µl of chloroform was added and the samples vortexed, followed by 250 µl of water and vortexed again. The mixture was made biphasic by centrifugation for 5 min at room temperature at 2000 g. The lower phase was transferred into new vials, dried under nitrogen and re-suspended into 600 µl of a 1∶2 chloroform:methanol solution. A portion (200 µl) was transferred into a new vial and all vials were dried under nitrogen. The lipids in the vials in which 200 µl were transferred were re-suspended in 250 µl of the standard diluent assay reagent and triglycerides measured following the manufacturer’s instructions. Three independent replicates were performed.

### Electrospray-mass spectrometry analysis

Lipid extracts from the vials in which 400 µl were transferred, were dissolved in 15 µl of choloroform:methanol (1∶2) and 15 µl of acetonitrile:iso-propanol:water (6∶7∶2) and analyzed with a Absceix 4000 QTrap, a triple quadrupole mass spectrometer equipped with a nanoelectrospray source.

Samples were delivered using either thin-wall nanoflow capillary tips or a Nanomate interface in direct infusion mode (∼125 nl/min). The lipid extracts were analyzed in both positive and negative ion modes using a capillary voltage of 1.25 kV. MS/MS scanning (daughter, precursor and neutral loss scans) were performed using nitrogen as the collision gas with collision energies between 35–90 V. Each spectrum encompasses at least 50 repetitive scans. Tandem mass spectra (MS/MS) were obtained with collision energies as follows: 35–45 V, PC/SM in positive ion mode, parent-ion scanning of m/z 184; 35–55 V, PI/IPC in negative ion mode, parent-ion scanning of m/z 241; 35–65 V, PE in negative ion mode, parent-ion scanning of m/z 196; 20–35 V, PS in negative ion mode, neutral loss scanning of m/z 87; and 40–90 V, for all glycerophospholipids (including PA, PG and cardiolipin) detected by precursor scanning for *m/z* 153 in negative ion mode. MS/MS daughter ion scanning was performed with collision energies between 35–90 V.

Assignment of phospholipid species is based upon a combination of survey, daughter, precursor and neutral loss scans, as well previous assignments [68]. The identity of phospholipid peaks was verified using the LIPID MAPS: Nature Lipidomics Gateway (www.lipidmaps.org).

### Identification and Quantification of Fatty acids

Full characterization and quantification of the fatty acids was conducted by conversion to the corresponding fatty acid methyl esters (FAME) followed by GC-MS analysis. Triplicate lipid extract aliquots were transferred to 2 ml glass vessels and spiked with an internal standard fatty acid C17∶0 (20 µl of 1 mM) and dried under nitrogen. Fatty acids were released by base hydrolysis using 500 µl of concentrated ammonia and 50% propan-1-ol (1∶1), followed by incubation for 5 h at 50°C.

After cooling the samples were evaporated to dryness with nitrogen and dried twice more from 200 µl of methanol:water (1∶1) to remove all traces of ammonia. The protonated fatty acids were now extracted by partitioning between 500 µl of 20 mM HCl and 500 µl of ether, the aqueous phase was re-extracted with fresh ether (500 µl) and the combined ether phases dried under nitrogen in a glass tube. The fatty acids were converted to methyl esters (FAME), by adding diazomethane (3×20 µl aliquots) to the dried residue, while on ice. After 30 min the samples were allowed to warm to RT and left to evaporate to dryness in a fume hood. The FAME products were dissolved in 10–20 µl dichloromethane and 1–2 µl analyzed by GC-MS on a Agilent Technologies (GC-6890N, MS detector-5973) with a ZB-5 column (30 M×25 mm×25 mm, Phenomenex), with a temperature program of at 70°C for 10 min followed by a gradient to 220°C at 5°C/min and held at 220°C for a further 15 min. Mass spectra were acquired from 50–500 amu. The identity of FAMEs was carried out by comparison of the retention time and fragmentation pattern with a FAME standards (Larodan) and bacterial FAME standards (Supelco).

### Glycogen and Protein Contents

Glycogen content was measured as described in Van Handel [69]. In each vial, 20 flies were kept and given spermidine or not for one week after which flies were then frozen in liquid nitrogen. Three flies were homogenized in 100 µl methanol and 50 µl of a saturated sodium sulphate (Sigma, 238597) solution. Samples were then centrifuged at 13000 g for 3 min and the supernatant removed. The pellet was then gently dissolved in 1 ml ultra pure water. Standards (10–50 mg glycogen, Sigma, G0885) were also prepared in 1 ml water. One hundred µl of the samples, standards or blank was then mixed gently with 3 ml of anthrone reagent (dilute 760 ml sulfuric acid into 300 ml water, add 150 mg anthrone (Sigma, 10740) to 100 ml of diluted sulfuric acid just before use), heated at 90°C for 20 min then cooled on ice. A portion of each sample (250 µl), standard and blank was transferred into a 96-well plate and the absorbance read at 630 nm. Three independent replicates were performed, each with 4 or 5 independent samples for each group.

For protein content, in each vial 20 flies were kept and given spermidine or not for one week after which flies were then frozen in liquid nitrogen. Twenty five flies were homogenized in 100 µl PBS and incubate at 70°C for 5 min. The samples were then diluted 1∶10 in PBS ad centrifuge at maximum speed for 3 min. Standards (0.5–2 mg/ml) were prepared with albumin (VWR, A16951). Five µl of homogenates, standards or blank were added to 250 µl of Bradford reagent (Sigma, B6916) into a 96-well plate and mix for 30 s. The absorbance was read at 570 nm after leaving the plates 30 min at room temperature. Three independent replicates were performed, each with 4 or 5 independent samples for each group.

### Food Intake

Food intake was measured as described in Libert et al. [23] in normal flies. Twenty flies were kept in each vial and given spermidine or not for one week. Then the flies were fed for 24 hrs a 5% sugar/yeast diet mixed with 0.1% of blue dye (food colorant, Dr Oetker). Flies were then frozen in liquid nitrogen. Five females or 8 males were homogenized in 200 µl PBS and centrifuged at 8000 g for 10 minutes. A portion of 150 µl was transferred into a 96-well plate and absorbance was measured at 630 nm. Standards were prepared from aliquots of dyed food to estimate the mass of food consumed. Two independent replicates were performed, each with 5 or 6 independent samples for each group.

### Climbing Activity

Flies were kept in individual vials in groups of ten. Once a week, the percentage of flies in each vial able to reach 8 cm in 10 s was measured until no fly could reach the threshold. Three independent replicates were performed. The experiment was performed in both normal and autophagy-deficient flies.

### Statistical Analyses

Data from glycogen and protein contents were expressed as content per mg of fly. Triglycerides content by colorimetric assay was expressed in microgram per fly. Data from triglycerides content by thin layer chromatography were weighted according to the weight of the flies in each sample. Data from food intake were expressed as intake per fly. The intensity data from the triglycerides content by thin layer chromatography were transformed into the inverse of the mean grey value to obtain a higher value for darker chromatography spots (the software ImageJ is set up so the darker the spot, the lower the mean grey value). For all experiments, the effect of spermidine was tested using non-parametric t-tests. Sex and genotype were analysed separately.

## Supporting Information

Figure S1 **Total fatty acid profile of males.** **A–D)** Identification and quantification of total fatty acid content from wild-type and atg7^−/−^ male flies. Fatty acids from male wild type flies left untreated (A) or fed 1 mM spermidine (C) and male atg7^−/−^ mutant flies left untreated (B) or fed 1 mM spermidine (D) were derivatised with diazomethane to the corresponding fatty acid methyl esters (FAMEs), together with an internal standard (C17∶0). The samples were analyzed by GC-MS and the retention times and fragmentation patterns, compared with FAME standards. 3 independent replicates were monitored for each group.
(TIF)

Figure S2 **Total fatty acid profile of females.** **A–D)** Identification and quantification of total fatty acid content from wild-type and atg7^−/−^ female flies. Fatty acids from female wild type flies left untreated (A) or fed 1 mM spermidine (C) and female atg7^−/−^ mutant flies left untreated (B) or fed 1 mM spermidine (D) were derivatised with diazomethane to the corresponding fatty acid methyl esters (FAMEs), together with an internal standard (C17∶0). The samples were analysed by GC-MS and the retention times and fragmentation patterns, compared with FAME standards. 3 independent replicates were monitored for each group.
(TIF)

Figure S3 **Phospholipid profile of males negative survey.** **A–D)** Negative ion ES-MS survey scans (600–1000 m/z) of total lipid extracts from male wild type (A, atg7^+/+^) and atg7^−/−^ (B) flies as well as from spermidine-fed male wild type (C, atg7^+/+^) and atg7^−/−^ (D) flies.
(TIF)

Figure S4 **Phospholipid profile of females negative survey.** **A–D)** Negative ion ES-MS survey scans (600–1000 m/z) of total lipid extracts from female wild type (A, atg7^+/+^) and atg7^−/−^ (B) flies as well as from spermidine-fed female wild type (C, atg7^+/+^) and atg7^−/−^ (D) flies.
(TIF)

Table S1 **Total fatty acids.** Fatty acids levels in males and females normal or autophagy-deficient, fed or not 1 mM spermidine for one week. We observe for normal males a decline in C14∶1, C16∶0, C18∶0, C20∶4 and C20∶3 and an increase in C18∶2, C20∶2 and C20∶1 upon spermidine treatment. In normal spermidine-fed females, we see a decline in C14∶1, C14∶0, C20∶3 and C20∶2 and an increase in C18∶2. Lack of autophagy triggers a decrease in the levels of fatty acids C16∶0 and C18∶0 in males. We observe in atg7^−/−^ males fed spermidine a decline in C18∶2 and C18∶0 and an increase in C14∶0, C18∶3, C20∶4, C20∶3 and C20∶2. In spermidine-fed at7^−/−^ females, we see a decline in C14∶1, C20∶4, C20∶3 and C20∶2 and an increase in C18∶2.
(DOCX)

Table S2 **Lipid species identified by mass spectrometry.**
(DOC)

## References

[1] Kenyon CJ (2010) The genetics of ageing. Nature 464: 504–512. 20336132 10.1038/nature08980

[2] Fontana L, Partridge L, Longo VD (2010) Extending healthy life span–from yeast to humans. Science 328: 321–326. 20395504 10.1126/science.1172539 PMC3607354

[3] Mattison JA, Roth GS, Beasley TM, Tilmont EM, Handy AM, et al (2012) Impact of caloric restriction on health and survival in rhesus monkeys from the NIA study. Nature 489: 318–321. 22932268 10.1038/nature11432 PMC3832985

[4] Baur JA, Pearson KJ, Price NL, Jamieson HA, Lerin C, et al (2006) Resveratrol improves health and survival of mice on a high-calorie diet. Nature 444: 337–342. 17086191 10.1038/nature05354 PMC4990206

[5] Miller RA, Harrison DE, Astle CM, Baur JA, Boyd AR, et al (2011) Rapamycin, but not resveratrol or simvastatin, extends life span of genetically heterogeneous mice. J Gerontol 66: 191–201. 10.1093/gerona/glq178 PMC3021372 20974732

[6] Harrison DE, Strong R, Sharp ZD, Nelson JF, Astle CM, et al (2009) Rapamycin fed late in life extends lifespan in genetically heterogeneous mice. Nature 460: 392–395. 19587680 10.1038/nature08221 PMC2786175

[7] Bjedov I, Toivonen JM, Kerr F, Slack C, Jacobson J, et al (2010) Mechanisms of life span extension by rapamycin in the fruit fly *Drosophila melanogaster* . Cell Metab 11: 35–46. 20074526 10.1016/j.cmet.2009.11.010 PMC2824086

[8] Harrison B, Tran TT, Taylor D, Lee SD, Min KJ (2010) Effect of rapamycin on lifespan in *Drosophila* . Geriatr Gerontol Int 10: 110–112. 20102391 10.1111/j.1447-0594.2009.00569.x

[9] Minois N, Carmona-Gutierrez D, Madeo F (2011) Polyamines in aging and disease. Aging 3: 716–732. 21869457 10.18632/aging.100361 PMC3184975

[10] Nishimura K, Shiina R, Kashiwagi K, Igarashi K (2006) Decrease in polyamines with aging and their ingestion from food and drink. J Biochem 139: 81–90. 16428322 10.1093/jb/mvj003

[11] Scalabrin G, Ferioli ME (1984) Polyamines in mammalian ageing: an oncological problem, too? A review. Mech Ageing Dev 26: 149–164. 6384679 10.1016/0047-6374(84)90090-3

[12] Pucciarelli S, Moreschini B, Micozzi D, De Fronzo GS, carpi FM, et al (2012) Spermidine and spermine are enriched in whole-blood of nona/centenarians. Rejuv Res 15: 590–595. 10.1089/rej.2012.1349 22950434

[13] Eisenberg T, Knauer H, Schauer A, Büttner S, Ruckenstuhl C, et al (2009) Induction of autophagy by spermidine promotes longevity. Nat Cell Biol 11: 1305–1314. 19801973 10.1038/ncb1975

[14] Soda K, Dobashi Y, Kano Y, Tsujinaka S (2009) Konishi (2009) Polyamine-rich food decreases age-associated pathology and mortality in aged mice. Exp Gerontol 44: 727–732. 19735716 10.1016/j.exger.2009.08.013

[15] Minois N, Carmona-Gutierrez D, Bauer MA, Rockenfeller P, Eisenberg T, et al (2012) Spermidine promotes stress resistance in *Drosophila melanogaster* through autophagy-dependent and –independent pathways. Cell Death Dis 3: e401. 23059820 10.1038/cddis.2012.139 PMC3481127

[16] Suppola S, Heikkinen S, Parkkinen JJ, Uusi-Oukari M, Korhonen VP, et al (2001) Concurrent overexpression of ornithine decarboxylase and spermidine/spermine N^1^-acetyltransferase further accelerates the catabolism of hepatic polyamines in transgenic mice. Biochem J 358: 343–348. 11513732 10.1042/0264-6021:3580343 PMC1222066

[17] Morselli E, Galluzzi L, Kepp O, Criollo A, Maiuri MC, et al (2009) Autophagy mediates pharmacological lifespan extension by spermidine and resveratrol. Aging 1: 961–970. 20157579 10.18632/aging.100110 PMC2815753

[18] Madeo F, Tavernarakis N, Kroemer G (2010) Can autophagy promote longevity? Nat Cell Biol 12: 842–846. 20811357 10.1038/ncb0910-842

[19] Markaki M, Tavernarakis N (2011) The role of autophagy in genetic pathways influencing ageing. Biogerontol 12: 377–386. 10.1007/s10522-011-9324-9 21347677

[20] Carmona-Gutierrez D, Bauer MA, Ring J, Knauer H, Eisenberg T, et al (2011) The propeptide of yeast cathepsin D inhibits programmed necrosis. Cell Death Dis 2: e161. 21593793 10.1038/cddis.2011.43 PMC3122122

[21] Giannakou ME, Partridge L (2007) Role of insulin-like signalling in *Drosophila* lifespan. Trends Biochem Sci 32: 180–188. 17412594 10.1016/j.tibs.2007.02.007

[22] Kapahi P, Chen D, Rogers AN, Katewa SD, Li PW, et al (2010) With TOR, less is more: a key role for the conserved nutrient-sensing TOR pathway in aging. Cell Metab 11: 453–465. 20519118 10.1016/j.cmet.2010.05.001 PMC2885591

[23] Libert S, Zwiener J, Chu X, Vanvoorhies V, Roman G, et al (2007) Regulation of *Drosophila* life span by olfaction and food-derived odors. Science 315: 1133–1137. 17272684 10.1126/science.1136610

[24] Shmookler Reis RJ, Xu L, Lee H, Chae M, Thaden JJ, et al (2011) Modulation of lipid biosynthesis contributes to stress resistance and longevity of *C. elegans mutants* . Aging 3: 125–144. 21386131 10.18632/aging.100275 PMC3082008

[25] O’Rourke EJ, Kuballa P, Xavier R, Ruvkun G (2013) ω-6 polyunsaturated fatty acids extend life span through the activation of autophagy. Genes & Dvpt 27: 429–440. 10.1101/gad.205294.112 PMC3589559 23392608

[26] Liao CY, Rikke BA, Johnson TE, Gelfond JAL, Diaz V, et al (2011) Fat maintenance is a predictor of the murine lifespan response to dietary restriction. Aging Cell 10: 629–639. 21388497 10.1111/j.1474-9726.2011.00702.x PMC3685291

[27] Katewa SD, Demontis F, Kolipinski M, Hubbard A, Gill MS, et al (2012) Intramyocellular fatty-acid metabolism plays a critical role in mediating responses to dietary restriction in *Drosophila melanogaster* . Cell Metab 16: 97–103. 22768842 10.1016/j.cmet.2012.06.005 PMC3400463

[28] Slack C, Werz C, Wieser D, Alic N, Foley A, et al (2010) Regulation of lifespan, metabolism, and stress responses by the *Drosophila* SH2B protein, Lnk. PLoS Genet 6: e1000881. 20333234 10.1371/journal.pgen.1000881 PMC2841611

[29] Minois N (2014) Molecular basis of the “anti-aging” effect of spermidine and other natural polyamines – A mini review. Gerontol 60: doi:10.1159/000356748. 10.1159/000356748 24481223

[30] Feany MB, Bender WW (2000) A Drosophila model of Parkinson’s disease. Nature 404: 394–398. 10746727 10.1038/35006074

[31] Minois N, Khazaeli AA, Curtsinger JW (2001) Locomotor activity as a function of age and life span in Drosophila melanogaster overexpressing hsp70. Exp Gerontol 36: 1137–1153. 11404055 10.1016/s0531-5565(00)00263-1

[32] Ledesm MD, Martin MG, Dotti CG (2012) Lipid changes in the aged brain: effect on synaptic function and neuronal survival. Prog Lipid Res 51: 23–25. 22142854 10.1016/j.plipres.2011.11.004

[33] Lapierre LR, Gelino S, Meléndez A, Hansen M (2011) Autophagy and lipid metabolism coordinately modulate life span in germline-less *C. elegans* . Curr Biol 21: 1507–1514. 21906946 10.1016/j.cub.2011.07.042 PMC3191188

[34] Gupta VK, Scheunemann L, Eisenberg T, Mertel S, Bhukel A, et al (2013) Restoring polyamines protects from age-induced memory impairment in an autophagy-dependent manner. Nature Neurosci 16: 1453–1460. 23995066 10.1038/nn.3512

[35] Gonzalez-Covarrubias V (2013) Lipidomics in longevity and healthy aging. Biogerontol Doi:10.1007/s10522-013-9450-7. 10.1007/s10522-013-9450-7 23948799

[36] Huang X, Withers BR, Dickson RC (2013) Sphingolipids and lifespan regulation. Biochim Biophys Acta http://dx.doi.org/10.1016/j.bbalip.2013.08.006. 10.1016/j.bbalip.2013.08.006 PMC3925463 23954556

[37] Ishii I, Ikeguchi Y, Mano H, Wada M, Pegg AE, et al (2012) Polyamine metabolism is involved in adipogenesis of 3T3-L1 cells. Amino Acids 42: 619–626. 21809076 10.1007/s00726-011-1037-5 PMC3266501

[38] Hyvönen MT, Koponen T, Weisell J, Pietilä M, Khomutov AR, et al. (2013) Spermidine promotes adipogenesis of 3T3-L1 cells by preventing interaction of ANP32 with HUR and PP2A. Biochem J doi:10.1043/BJ20130263. 10.1042/BJ20130263 23672317

[39] Choi YH, Park HY (2012) Anti-inflammatory effects of spermidine in lipopolysaccharide-stimulated BV2 microglial cells. J Biomed Sci 19: doi:10.1186/1423-0127-19-31. 10.1186/1423-0127-19-31 PMC3320531 22433014

[40] Inoue K, Tsutsui H, Akatsu H, Hashizume Y, Matsukawa N, et al. (2013) Metabolic profiling of Alzheimer’s disease brains. Sci Rep 3: doi:10.1038/srep02364. 10.1038/srep02364 PMC3734482 23917584

[41] Lee NK, MacLean HE (2011) Polyamines, androgens, and skeletal muscle hypertrophy. J Cell Physiol 226: 1453–1460. 21413019 10.1002/jcp.22569

[42] Luchessi AD, Cambiaghi TD, Hirabara SM, Lambertucci RH, Silveira LR, et al (2009) Involvement of eukaryotic translation initiation factor 5A (eIF5A) in skeletal muscle stem cell differentiation. J Cell Physiol 218: 480–489. 19006180 10.1002/jcp.21619

[43] Herndon LA, Schmeissner PJ, Dudaronek JM, Brown PA, Listner KM, et al (2002) Stochastic and genetic factors influence tissue-specific decline in ageing C. elegans. Nature 419: 808–814. 12397350 10.1038/nature01135

[44] Song W, Ren D, Li W, Jiang L, Cho KW, et al (2010) SH2B regulation of growth, metabolism and longevity in both insects and mammals. Cell Metab 11: 427–437. 20417156 10.1016/j.cmet.2010.04.002 PMC2881875

[45] Poon PC, Kuo TH, Linford NJ, Roman G, Pletcher SD (2010) Carbon dioxide sensing modulates lifespan and physiology in *Drosophila* . PLoS Biol 8: e1000356. 20422037 10.1371/journal.pbio.1000356 PMC2857880

[46] Banerjee KK, Ayyub C, Sengupta S, Kolthur-Seetharam U (2012) dSir2 deficiency in the fat body, but not muscles, affects systemic insulin signalling, fat mobilization and starvation survival in flies. Aging 4: 206–223. 22411915 10.18632/aging.100435 PMC3348481

[47] Lee SH, Lee SK, Paik D, Min KJ (2012) Overexpression of fatty-acid-β-oxidation-related genes extends the lifespan of *Drosophila melanogaster*. Oxid Med Cell Longev 854502. 10.1155/2012/854502 PMC3446750 22997544

[48] Rustici G, Kolesnikov N, Brandizi M, Burdett T, Dylag M, et al (2013) ArrayExpress update-trends in database growth and links to data analysis tools. Nucl Acids Res 41: D987–D990. 23193272 10.1093/nar/gks1174 PMC3531147

[49] Unger RH, Clark GO, Scherer PE, Orci L (2010) Lipid homeostasis, lipotoxicity and the metabolic syndrome. Biochim Biophys Acta 1801: 209–214. 19948243 10.1016/j.bbalip.2009.10.006

[50] Singh R, Kaushik S, Wang Y, Xiang Y, Novak I, et al (2009) Autophagy regulates lipid metabolism. Nature 458: 1131–1135. 19339967 10.1038/nature07976 PMC2676208

[51] Carvalho M, Sampio JL, Palm W, Brakatschk M, Eaton S, et al. (2012) Effects of diet and development on the *Drosophila* lipidome. Mol Syst Biol 8: doi:10.1038/msb.2012.29. 10.1038/msb.2012.29 PMC3421444 22864382

[52] Eckmann J, Eckert SH, Leuner K, Muller WE, Eckert GP (2013) Mitochondria: mitochondrial membranes in brain ageing and neurodegeneration. Int J Biochem Cell Biol 45: 76–80. 22743330 10.1016/j.biocel.2012.06.009

[53] Parisi M, Li R, Oliver B (2011) Lipid profiles of female and male Drosophila. BMC Res Notes 4: doi:10.1186/1756-0500-4-198. 10.1186/1756-0500-4-198 PMC3146437 21676256

[54] Baerga R, Zhang Y, Chen PH, Goldman S, Jin S (2009) Targeted deletion of autophagy-related 5 (atg5) impairs adipogenesis in a cellular model and in mice. Autophagy 5: 1118–1130. 19844159 10.4161/auto.5.8.9991 PMC2873687

[55] Zhang Y, Goldman S, Baerga R, Zhao Y, Komatsu M, et al (2009) Adipose-specific deletion of autophagy-related gene 7 (atg7) in mice reveals a role in adipogenesis. Proc Natl Acad Sci USA 106: 19860–19865. 19910529 10.1073/pnas.0906048106 PMC2785257

[56] Singh R, Xiang Y, Wang Y, Baikati K, Cuervo AM, et al (2009) Autophagy regulates adipose mass and differentiation in mice. J Clin Invest 119: 3329–3339. 19855132 10.1172/JCI39228 PMC2769174

[57] Morselli E, Mariño G, Bennetzen MV, Eisenberg T, Megalou E, et al (2011) Spermidine and resveratrol induce autophagy by distinct pathways converging on the acetylproteome. J Cell Biol 192: 615–629. 21339330 10.1083/jcb.201008167 PMC3044119

[58] Bennetzen MV, Marino G, Pultz D, Morselli E, Faergeman NJ, et al (2012) Phosphoproteomic analysis of cells treated with longevity-related autophagy inducers. Cell Cycle 11: 1827–1840. 22517431 10.4161/cc.20233

[59] Rajeeve V, Pearce W, Cascante M, Vanhaesebroeck B, Cutillas PR (2013) Polyamine production is downstream and upstream of oncogenic PI3K signalling and contributes to tumour cell growth. Biochem J 450: 619–628. 23330613 10.1042/BJ20121525

[60] Choi YH, Park HY (2012) Anti-inflammatory effects of spermidine in lipopolysaccharide-stimulated BV2 microglial cells. J Biomed Sci doi:10.1186/1423-0127-19-31. 10.1186/1423-0127-19-31 PMC3320531 22433014

[61] Wang J, Whiteman MW, Lian H, Wang G, Singh A, et al (2009) A non-canonical MEK/ERK signalling pathway regulates autophagy via regulating Beclin 1. J Biol Chem 284: 21412–21424. 19520853 10.1074/jbc.M109.026013 PMC2755866

[62] Stark F, Pfannstiel J, Klaiber I, Raabe T (2011) Protein kinase CK2 links polyamine metabolism to MAPK signalling in *Drosophila* . Cell Signal 23: 876–882. 21262350 10.1016/j.cellsig.2011.01.013

[63] Cheng L, Sun RR, Wang FY, Peng Z, Kong FL, et al (2012) Spermidine affects the transcriptome responses to high temperature stress in ripening tomato fruit. J Zhejiang Univ Sci B 13: 283–297. 22467370 10.1631/jzus.B1100060 PMC3323944

[64] Lin YR, Kim K, Yang Y, Ivessa A, Sadoshima J, et al (2011) Regulation of longevity by regulator of G-protein signalling protein, Loco. Aging Cell 10: 438–447. 21255223 10.1111/j.1474-9726.2011.00678.x

[65] Lin YR, Parikh H, Park Y (2011) Loco signalling pathway in longevity. Small GTPases 2: 158–161. 21776417 10.4161/sgtp.2.3.16390 PMC3136946

[66] Juhász G, Érdi B, Sass M, Neufeld TP (2007) Atg7-dependent autophagy promotes neuronal health, stress tolerance, and longevity but is dispensable for metamorphosis in *Drosophila* . Genes & Dvpt 21: 3061–3066. 10.1101/gad.1600707 PMC2081972 18056421

[67] Al-Anzi B, Sapin V, Waters C, Zinn K, Wyman RJ, et al (2009) Obesity-blocking neurons in *Drosophila* . Neuron 63: 329–341. 19679073 10.1016/j.neuron.2009.07.021 PMC2742587

[68] Richmond GS, Gibellini F, Young SA, Major L, Denton H, et al (2010) Lipidomic analysis of bloodstream and procyclic form *Trypanosoma brucei* . Parasitol 137: 1357–1392. 10.1017/S0031182010000715 PMC3744936 20602846

[69] Van Handel E (1965) Microseparation of glycogen, sugars, and lipids. Anal Biochem 11: 266–271. 4378807 10.1016/0003-2697(65)90014-x

