# Supplementary figures and images for: Spermidine Feeding Decreases Age-Related Locomotor Activity Loss and Induces Changes in Lipid Composition
Source: PLoS One. 2014 Jul 10;9(7):e102435. doi: 10.1371/journal.pone.0102435 (PMC4092136; doi:10.1371/journal.pone.0102435)

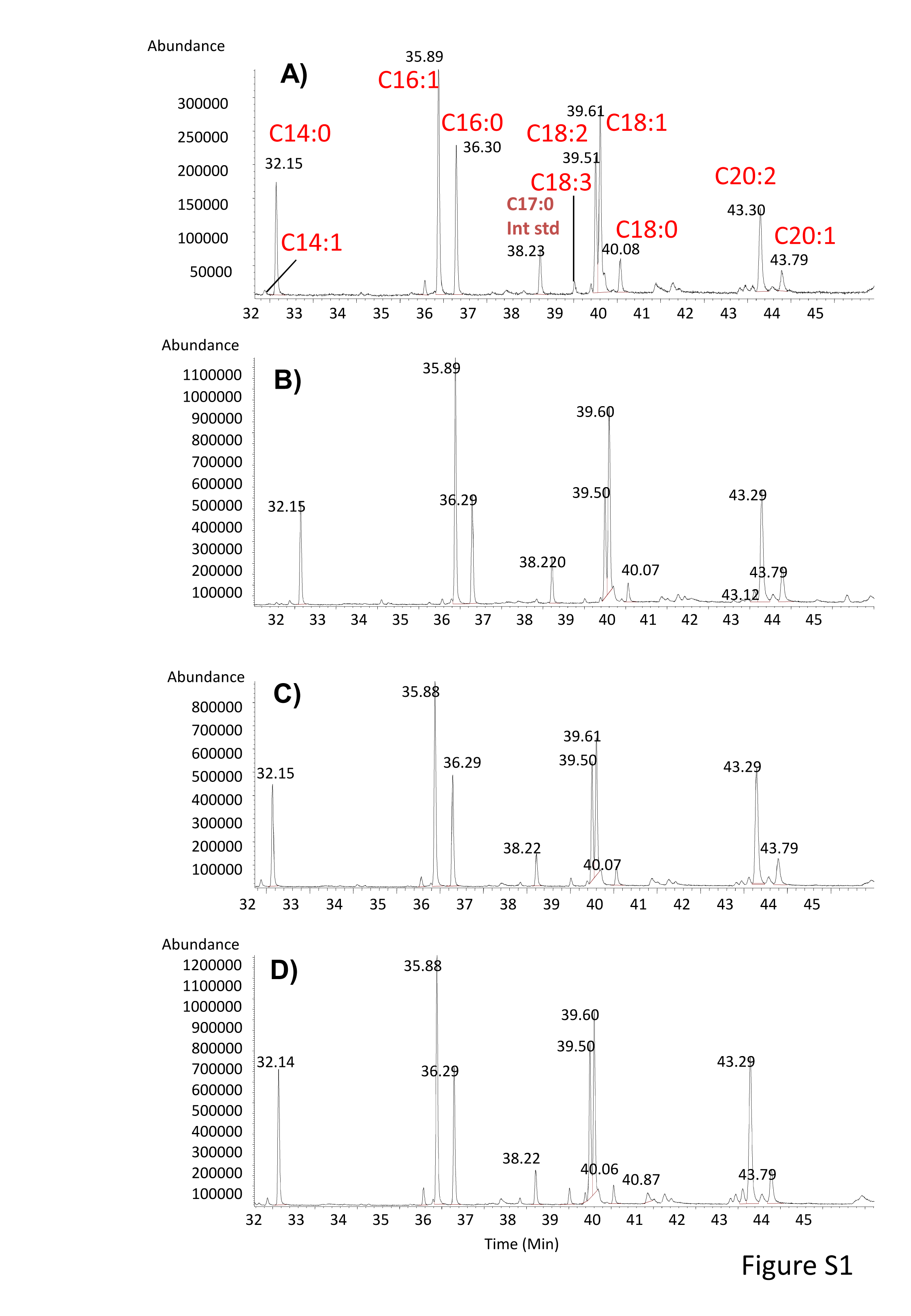

Supplement: Figure S1 — Total fatty acid profile of males. A–D) Identification and quantification of total fatty acid content from wild-type and atg7−/− male flies. Fatty acids from male wild type flies left untreated (A) or fed 1 mM spermidine (C) and male atg7−/− mutant flies left untreated (B) or fed 1 mM spermidine (D) were derivatised with diazomethane to the corresponding fatty acid methyl esters (FAMEs), together with an internal standard (C17∶0). The samples were analyzed by GC-MS and the retention times and fragmentation patterns, compared with FAME standards. 3 independent replicates were monitored for each group. (TIF) [file pone.0102435.s001.tif]

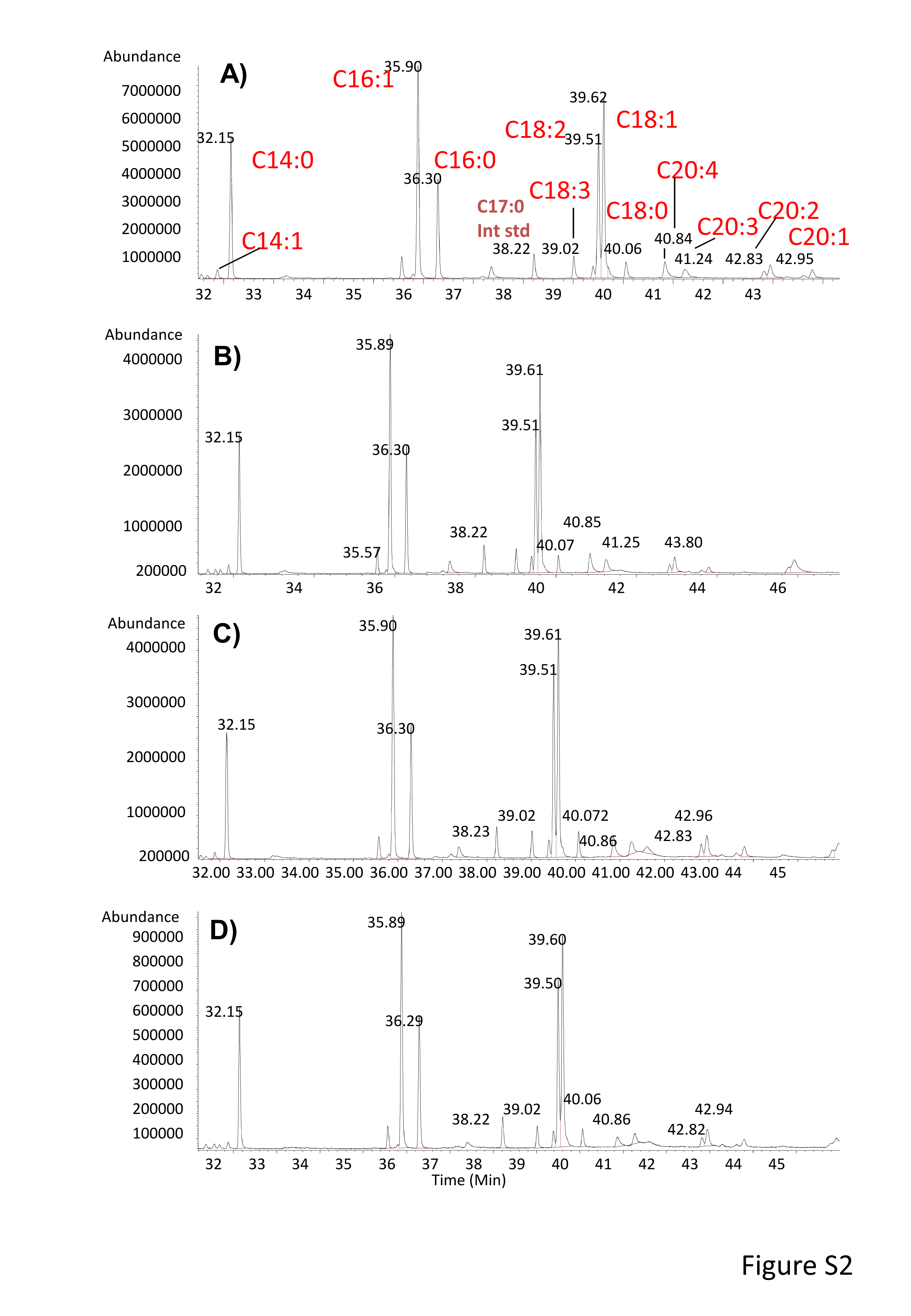

Supplement: Figure S2 — Total fatty acid profile of females. A–D) Identification and quantification of total fatty acid content from wild-type and atg7−/− female flies. Fatty acids from female wild type flies left untreated (A) or fed 1 mM spermidine (C) and female atg7−/− mutant flies left untreated (B) or fed 1 mM spermidine (D) were derivatised with diazomethane to the corresponding fatty acid methyl esters (FAMEs), together with an internal standard (C17∶0). The samples were analysed by GC-MS and the retention times and fragmentation patterns, compared with FAME standards. 3 independent replicates were monitored for each group. (TIF) [file pone.0102435.s002.tif]

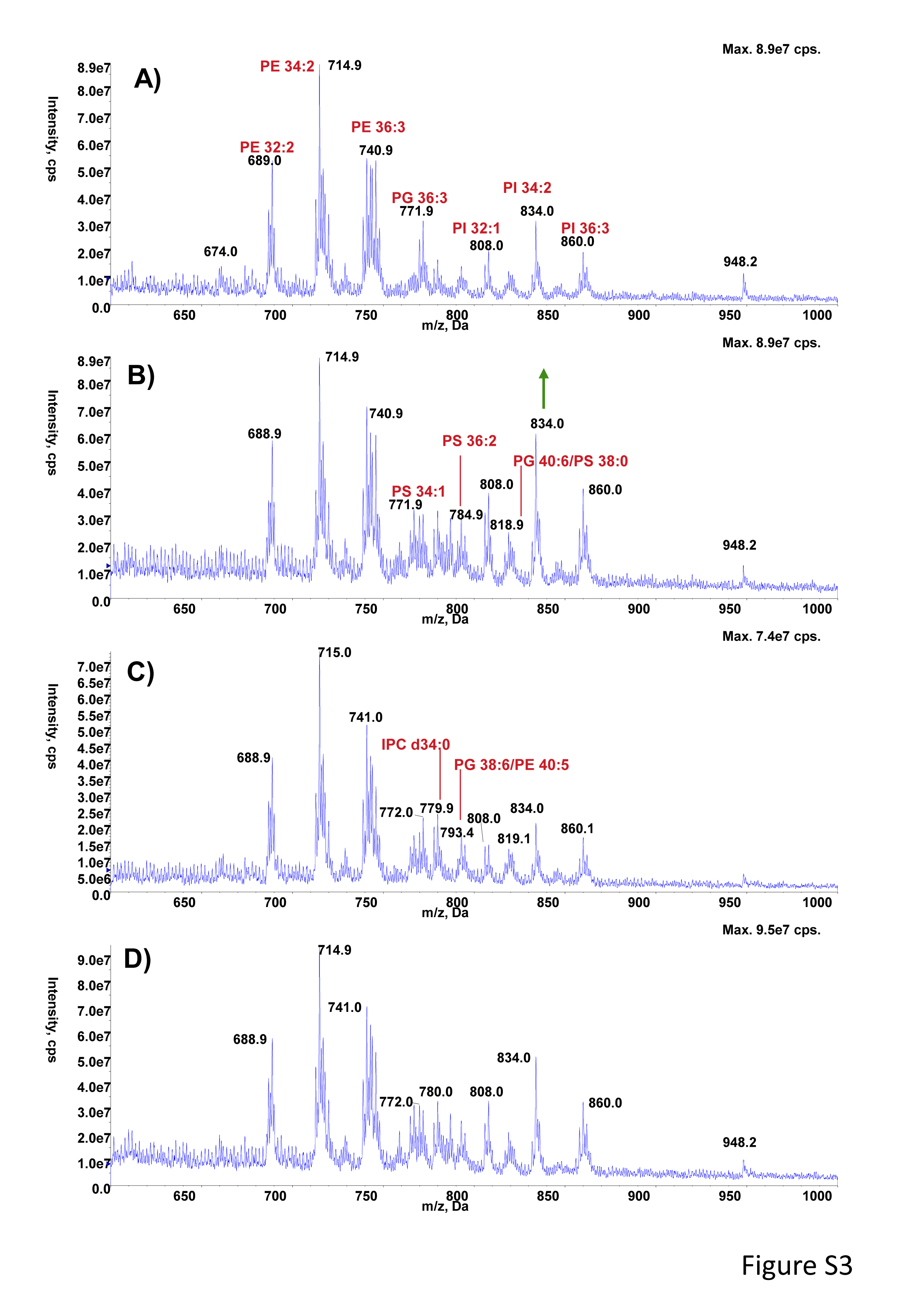

Supplement: Figure S3 — Phospholipid profile of males negative survey. A–D) Negative ion ES-MS survey scans (600–1000 m/z) of total lipid extracts from male wild type (A, atg7+/+) and atg7−/− (B) flies as well as from spermidine-fed male wild type (C, atg7+/+) and atg7−/− (D) flies. (TIF) [file pone.0102435.s003.tif]

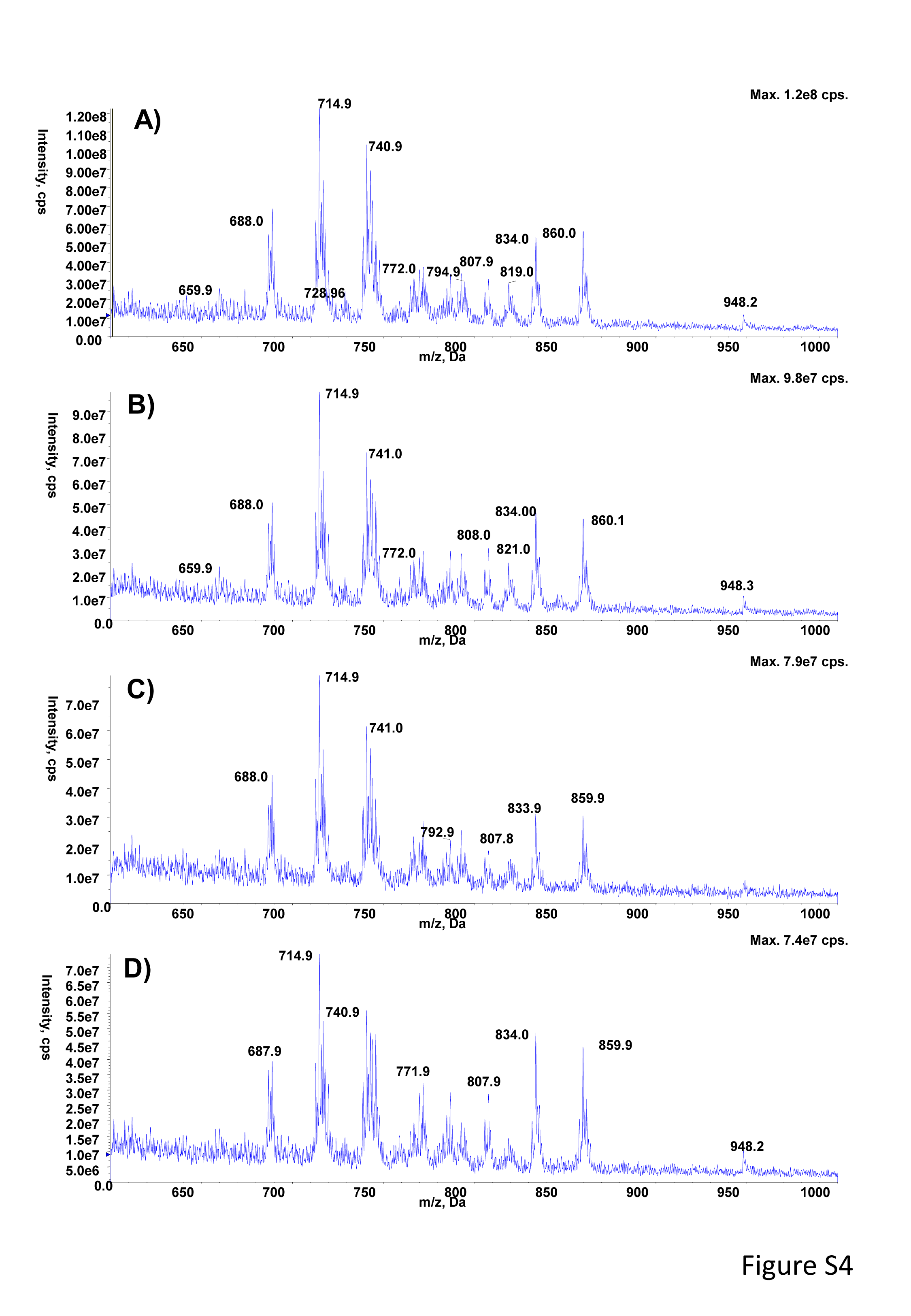

Supplement: Figure S4 — Phospholipid profile of females negative survey. A–D) Negative ion ES-MS survey scans (600–1000 m/z) of total lipid extracts from female wild type (A, atg7+/+) and atg7−/− (B) flies as well as from spermidine-fed female wild type (C, atg7+/+) and atg7−/− (D) flies. (TIF) [file pone.0102435.s004.tif]
