# Supplementary material for: Spermidine Feeding Decreases Age-Related Locomotor Activity Loss and Induces Changes in Lipid Composition
Source: PLoS One. 2014 Jul 10;9(7):e102435. doi: 10.1371/journal.pone.0102435 (PMC4092136; doi:10.1371/journal.pone.0102435)
Supplement: Table S1 — Total fatty acids. Fatty acids levels in males and females normal or autophagy-deficient, fed or not 1 mM spermidine for one week. We observe for normal males a decline in C14∶1, C16∶0, C18∶0, C20∶4 and C20∶3 and an increase in C18∶2, C20∶2 and C20∶1 upon spermidine treatment. In normal spermidine-fed females, we see a decline in C14∶1, C14∶0, C20∶3 and C20∶2 and an increase in C18∶2. Lack of autophagy triggers a decrease in the levels of fatty acids C16∶0 and C18∶0 in males. We observe in atg7−/− males fed spermidine a decline in C18∶2 and C18∶0 and an increase in C14∶0, C18∶3, C20∶4, C20∶3 and C20∶2. In spermidine-fed at7−/− females, we see a decline in C14∶1, C20∶4, C20∶3 and C20∶2 and an increase in C18∶2. (DOCX) [file pone.0102435.s005.docx]

Table S1A: Total fatty acids

| FAME  (Retention time –min) | Atg7^+/+^ males | Atg7^-/-^ males | Atg7^+/+^ males + 1mM spermidine | Atg7^-/-^ males + 1mM spermidine | Atg7^+/+^ females | Atg7^-/-^ females | Atg7^+/+^ females + 1mM spermidine | Atg7^-/-^ females + 1mM spermidine |
| --- | --- | --- | --- | --- | --- | --- | --- | --- |
| C14:1 (31.8) | 0.27 ± 0.05 | 0.1 ± 0.05 | 0.1 ± 0.05 | 0.1 ± 0.05 | 0.9 ± 0.05 | 0.9 ± 0.05 | 0.6 ± 0.05 | 0.3 ± 0.05 |
| C14:0 (32.1) | 10.5 ± 0.34 | 9.7 ± 0.62 | 10.6 ± 0.56 | 11.1 ± 1.05 | 13.8 ± 0.74 | 13.0 ± 0.36 | 11.1 ± 0.39 | 13.1 ± 0.22 |
| C16:1 (35.8) | 21.7 ± 0.79 | 23.4 ± 1.43 | 21.7 ± 1.15 | 20.6 ± 1.66 | 23.1 ± 1.25 | 23.1 ± 1.06 | 21.8 ± 0.58 | 22.9 ± 0.46 |
| C16:0 (36.3) | 14.2 ± 0.98 | 10.6 ± 0.88 | 11.7 ± 0.95 | 10.9 ± 1.02 | 10.7 ± 0.49 | 11.9 ± 0.62 | 11.8 ± 0.46 | 12.5 ± 0.32 |
| C18:3 (39.0) | 0.5 ± 0.05 | 0.2 ± 0.05 | 0.4 ± 0.05 | 1.4 ± 0.17 | 2.5 ± 0.13 | 2.5 ± 0.05 | 2.5 ± 0.29 | 2.3 ± 0.08 |
| C18:2 (39.5) | 12.0 ± 0.69 | 16.0 ± 1.42 | 12.5 ± 1.24 | 12.8 ± 0.82 | 15.2 ± 0.21 | 14.6 ± 0.46 | 17.0 ± 0.91 | 16.5 ± 0.64 |
| C18:1 (39.6) | 21.2 ± 1.19 | 18.4 ± 0.90 | 14.8 ± 1.08 | 15.0 ± 0.77 | 20.8 ± 0.71 | 21.9 ± 0.58 | 22.7 ± 0.95 | 23.8 ± 0.83 |
| C18:0 (40.1) | 3.6 ± 0.59 | 2.2 ± 0.13 | 2.5 ± 0.12 | 1.6 ± 0.16 | 2.0 ± 0.05 | 1.8 ± 0.05 | 2.5 ± 0.17 | 1.9 ± 0.13 |
| C20:4 (40.8) | 1.7 ± 0.24 | 0.3 ± 0.05 | 0.4 ± 0.05 | 1.2 ± 0.07 | 3.3 ± 0.07 | 3.6 ± 0.07 | 3.3 ± 0.11 | 2.0 ± 0.16 |
| C20:3 (41.2) | 1.7 ± 0.17 | 0.2 ± 0.05 | 0.2 ± 0.05 | 0.5 ± 0.05 | 2.1 ± 0.05 | 2.6 ± 0.07 | 1.5 ± 0.10 | 1.2 ± 0.08 |
| C20:2 (42.9) | 11.7 ± 0.81 | 17.8 ± 1.35 | 19.8 ± 1.37 | 19.6 ± 1.06 | 4.0 ± 0.11 | 3.3 ± 0.08 | 3.3 ± 0.23 | 2.8 ± 0.35 |
| C20:1 (43.8) | 2.9 ± 0.33 | 6.1± 0.57 | 5.1 ± 0.13 | 5.2 ± 0.68 | 1.6 ± 0.18 | 0.9 ± 0.05 | 1.7 ± 0.12 | 0.8 ± 0.05 |
| Relative total fatty acid content | 100 ± 4.7 % | 99.5 ± 3.9 % | 134.3 ± .2 % | 168.1 ± 7.9 % | 100 ± 4.9 %  (173 ± 8.5 % relative to male control) | 97.0 ± 6.6 % | 95.1 ± 5.1 % | 85.8 ± 3.3 % |

Table S1B: Fatty acids derived from TAG
