# Supplementary material for: Spermidine Feeding Decreases Age-Related Locomotor Activity Loss and Induces Changes in Lipid Composition
Source: PLoS One. 2014 Jul 10;9(7):e102435. doi: 10.1371/journal.pone.0102435 (PMC4092136; doi:10.1371/journal.pone.0102435)
Supplement: Table S2 — Lipid species identified by mass spectrometry. (DOC) [file pone.0102435.s006.doc]

**Supplementary Table 2: Mass spectrometric analysis of phospholipid species in *Drosophila melanogaster***

1. The molecular species detected within a series are grouped. The peaks containing the most abundant species are in bold,
2. Mass over charge values (survey scan) ± 0.1 m/z were obtained as described in Materials and Methods.
3. Peak identities refer to total number of carbon atoms and double bonds.
4. Precise fatty acyl constituents and their positions are not discernable in positive ion mode; however, where possible, the most likely fatty acid candidates for the *sn*-1 and *sn*-2 constituents of the molecular species presented were deduced from the available literature. Only the principal component is given, in some instances as many as 4 individual species, with different combinations of acyl chains result in the same total number of carbon atoms and double bonds.

**PHOSPHATIDYLCHOLINE SPECIES**

| Peak^a^ | *m/z*^b^ |  | Lipid component^c^ | Principal component^d^, |
| --- | --- | --- | --- | --- |
| 1 | 686.3 |  | 28:1 | 14:0/14:1 |
| 2 | 686.3 |  | 28:0 | 14:0/14:0 |
| 3 | 702.5 |  | 30:2 | 16:1/14:1 |
| **4** | **704.5** |  | **30:1** | **16:0/14:1** |
| **5** | **706.5** |  | **30:0** | **16:0/14:0** |
| **6** | **730.6** |  | **32:2** | **16:1/16:1** |
| **7** | **732.6** |  | **32:1** | **16:0/16:1** |
| 8 | 734.58 |  | 32:0 | 16:0/16:0 |
| 9 | 754.55 |  | 34:4 | 16:1/18:3 |
| **10** | **756.57** |  | **34:3** | **16:1/18:2** |
| **11** | **758.59** |  | **34:2** | **16:0/18:2** |
| 12 | 760.59 |  | 34:1 | 16:0/18:1 |
| 13 | 762.62 |  | 34:0 | 16:0/18:0 |
| 14 | 778.5 |  | 36:6 | 18:3/18:3 |
| **15** | **780.5** |  | **36:5** | **18:2/18:3** |
| **16** | **782.5** |  | **36:4** | **18:2/18:2** |
| 17 | 784.6 |  | 36:3 | 18:1/18:2 |
| 18 | 786.6 |  | 36:2 | 18:0/18:2 & 18:1/18:1 |
| 19 | 788.6 |  | 36:1 | 18:0/18:1 |
| 20 | 790.6 |  | 36:0 | 18:0/18:0 |
| 21 | 808.53 |  | 38:5 | 18:3/20:2 |
| 22 | 810.58 |  | 38:4 | 18:2/20:2 |
| 23 | 812.60 |  | 38:3 | 18:1/20:2 |
| **24** | **814.62** |  | **38:2** | **18:2/20:0** |
| **25** | **816.63** |  | **38:1** | **18:1/20:0** |
| 26 | 818.67 |  | 38:0 | 18:0/20:0 |
| 27 | 838.62 |  | 40:4 | 20:2/20:2 |
| 28 | 840.65 |  | 40:3 | 20:1/20:2 |
| 29 | 842.68 |  | 40:2 | 20:0/20:2 |
| 30 | 844.72 |  | 40:1 | 18:1/20:0 |
| 31 | 846.73 |  | 40:0 | 18:0/22:0 |
|  |  |  |  |  |

**PHOSPHATIDYLETHANOLAMINE SPECIES**

| Peak^a^ | *m/z*^b^ | Lipid component^c^ | Principal component^d^ |
| --- | --- | --- | --- |
| 1 | 634.4 | 28:0 | 14:0/14:0 |
| 2 | 656.4 | 30:3 | 14:1/16:2 |
| 3 | 658.3 | 30:2 | 14:0/16:2 |
| 4 | 660.4 | 30:1 | 14:0/16:1 |
| 5 | 662.4 | 30:0 | 14:0/16:0 |
| 6 | 684.4 | 32:3 | 16:1/16:2 |
| **7** | **686.4** | **32:2** | **16:1/16:1** |
| **8** | **688.4** | **32:1** | **16:0/16:1** |
| 9 | 690.4 | 32:0 | 16:0/16:0 |
| 13 | 710.4 | 34:4 | 16:1/18:3 |
| **14** | **712.4** | **34:3** | **16:1/18:2** |
| **15** | **714.4** | **34:2** | **16:0/18:2** |
| **16** | **716.4** | **34:1** | **16:0/18:1** |
| 17 | 718.4 | 34:0 | 16:0/18:0 |
| 23 | 738.4 | 36:4 | 18:2/18:2 |
| **24** | **740.4** | **36:3** | **18:1/18:2** |
| **25** | **742.4** | **36:2** | **18:0/18:2** |
| **26** | **744.5** | **36:1** | **18:0/18:1** |
| 27 | 746.5 | 36:0 | 18:0/18:0 |
| 30 | 762.5 | 38:6 | 18:2/20:4 |
| 31 | 764.5 | 38:5 | 18:1/20:4 |
| 32 | 766.4 | 38:4 | 18:0/20:4 |
| 33 | 768.4 | 38:3 | 18:1/20:2 |
| 34 | 770.4 | 38:2 | 18:0/20:2 |
| 35 | 772.4 | 38:1 | 18:0/20:1 |
| 36 | 794.4 | 40:4 | 20:2/20:2 |
| 37 | 796.4 | 40:3 | 20:1/20:2 |
| 38 | 798.4 | 40:2 | 20:0/20:2 |

**PHOSPHATIDYLGLYCEROL SPECIES**

| Peak^a^ | *m/z*^b^ | Lipid component^c^ | Principal component^d^ |
| --- | --- | --- | --- |
| 1 | 718.4 | 32:2 | 16:1/16:1 |
|  | 720.4 | 32:1 | 16:0/16:1 |
| 2 | 722.4 | 32:0 | 16:0/16:0 |
| 3 | 710.4 | 34:4 | 16:1/18:3 |
| 4 | 712.4 | 34:3 | 16:1/18:2 |
| **5** | **746.5** | **34:2** | **16:0/18:2** |
| 6 | 748.5 | 34:1 | 16:0/18:1 |
| 7 | 750.5 | 34:0 | 16:0/18:0 |
| 8 | 768.4 | 36:5 | 14:0/22:5 |
| 9 | 770.4 | 36:4 | 18:2/18:2 |
| **10** | **772.5** | **36:3** | **18:1/18:2** |
| **11** | **774.5** | **36:2** | **18:0/18:2** |
| 12 | 776.5 | 36:1 | 18:0/18:1 |
| 13 | 778.5 | 36:0 | 18:0/18:0 |
| 14 | **794.4** | **38:6** | **18:2/20:4** |
| 15 | 796.4 | 38:5 | 18:3/20:2 |
| 16 | 798.4 | 38:4 | 18:2/20:2 |
| 17 | 800.4 | 38:3 | 18:1/20:2 |
| 18 | 802.4 | 38:2 | 18:0/20:2 |
| 19 | 804.4 | 38:1 | 18:1/20:0 |
| 20 | **820.3** | **40:6** | **20:2/20:4** |
| 21 | 824.3 | 40:4 | 20:2/20:2 |

**PHOSPHATIDYLSERINE SPECIES**

| Peak | *m/z* | Lipid component^c^ | Principal component^d^ |
| --- | --- | --- | --- |
|  |  |  |  |
| 1 | 759.5 | 34:2 | 16:0/18:2 |
| 2 | 761.5 | 34:1 | 16:0/18:1 |
| 3 | 763.5 | 34:0 | 16:0/18:0 |
| 4 | 783.5 | 36:4 | 18:2/18:2 |
| 5 | 785.5 | 36:3 | 18:1/18:2 |
| **6** | **787.5** | **36:2** | **18:0/18:2** |
| 7 | 789.5 | 36:1 | 18:0/18:1 |
| 8 | 809.5 | 38:5 | 18:1/20:4 |
| 9 | 811.5 | 38:4 | 18:0/20:4 |
| 10 | 813.5 | 38:3 | 18:1/20:2 |
| 11 | 815.5 | 38:2 | 18:2/20:0 |
| 12 | 817.5 | 38:1 | 18:1/20:0 |
| **13** | **819.5** | **38:0** | **18:0/20:0** |
|  |  |  |  |

**PHOSPHATIDYLIONSITOL SPECIES**

| Peak^a^ | *m/z*^b^ | Lipid component^c^ | Principal component^d^ |
| --- | --- | --- | --- |
| 7 | 806.4 | 32:2 | 16:1/16:1 |
| **8** | **808.4** | **32:1** | **16:0/16:1** |
| 9 | 810.4 | 32:0 | 16:0/16:0 |
| 14 | 832.4 | 34:3 | 16:1/18:2 |
| **15** | **834.5** | **34:2** | **16:0/18:2** |
| 16 | 836.5 | 34:1 | 16:0/18:1 |
| 17 | 838.5 | 34:0 | 16:0/18:0 |
| 22 | 856.4 | 36:5 | 18:2/18:3 |
| 23 | 858.4 | 36:4 | 18:2/18:2 |
| **24** | **860.5** | **36:3** | **18:1/18:2** |
| 25 | 862.5 | 36:2 | 18:0/18:2 |
| 26 | 864.5 | 36:1 | 18:0/18:1 |
| 27 | 866.5 | 36:0 | 18:0/18:0 |

**CARDIOLIPIN SPECIES**

| Peak | *m/z* | Lipid component^c^ | Principal component^d^ |
| --- | --- | --- | --- |
|  |  |  |  |
| 1 | 1344.8 | 64:4-2 | 16:1 |
| 2 | 1370.9 | 66:6-4 | 16:1 |
| 3 | 1396.9 | 68:6-4 | 18:1 |
| **4** | **1422.9** | **70:8-4** | **18:2** |
| **5** | **1448.9** | **72:8-4** | **18:2** |
| **6** | **1475.0** | **74:8-4** | **18:2** |

**ETHANOLAMINE-PHOSPHATE-CERAMIDE (EPC) SPECIES**

| Peak | *m/z* | Lipid component^c^ | Principal component^d^ |
| --- | --- | --- | --- |
| **1** | 632.4 | d32:1 | d32:1 |
| 2 | 634.4 | d32:0 | d32:0 |
| **3** | **660.4** | **d34:1** | **d34:1** |
| **4** | **662.4** | **d34:0** | **d34:0** |
| 5 | 688.5 | d36:1 | d36:1 |
| 6 | 690.5 | d36:0 | d36:0 |
| 7 | 716.5 | d38:1 | d38:1 |
| 8 | 718.6 | d38:0 | d38:0 |
|  |  |  |  |
